# Evaluation of Sperm Impairing Factor from *Serratia marcescens* as Male Contraceptive in Mouse Model

**DOI:** 10.1155/2019/9430964

**Published:** 2019-10-30

**Authors:** Aditi Chauhan, Vijay Prabha

**Affiliations:** Department of Microbiology, Panjab University, Chandigarh-160014, India

## Abstract

The present study was carried out to assess the contraceptive efficacy of sperm agglutinating factor (SAF) isolated from *Serratia marcescens*, in male Balb/c mice. Mice were administered via an intratesticular route with different concentrations of SAF, *viz.*, 10, 50, 100, 200, or 400 *µ*g, in the right testis only which served as a test while the left side served as control except otherwise stated. Mice were sacrificed on day 3, 7, 14, 21, 30, 45, 60, and 90 after administration, and results in terms of change in body weight, seminal parameters, tissue somatic indices (TSI), hematological parameters, serum level of testosterone, lipid peroxidation, and histology were studied. The body weight and TSI remained unaffected in all the experimental groups. In case of seminal parameters, the right testis treated with 10 *μ*g, 50 *μ*g, 100 *μ*g, 200 *μ*g, or 400 *μ*g of SAF showed azoospermia up to day 7, 14, 21, 45, and 90, respectively. The hematological indices, aspartate aminotransferase (AST), and alanine aminotransferase (ALT) were found to be unaltered when the group receiving SAF (test) was compared with the groups receiving phosphate buffer saline (control) in the right testis; however, the treatment had a negative effect on the serum level of testosterone. It also affected the oxidative status of the right testis. Furthermore, histological studies revealed hypospermatogenesis and alterations in the seminiferous tubules which included intraepithelial vacuolation and exfoliation in the right side as compared to the left side. Thus, the results suggest that SAF (400 *µ*g) causes suppression of spermatogenesis, without causing apparent toxic effects.

## 1. Introduction

The world's population is now odds-on to grow ever-higher for the rest of the century, presenting grave difficulties for food supplies, healthcare, and social cohesion. Contraception is considered to be the key answer to this. Although commendable progress has been made in developing highly efficient, user-friendly, safe, and reversible contraceptive methods for females, progress in case of males is creeping at a slow pace. Surprisingly, even today, the contraceptive choices available for men do not differ much from the ones available in the first century. Until today, condom and vasectomy have been the major methods employed for contraception in males. While investigating other promising methods for male contraception, several methods have been identified using hormonal approaches. With various lacunae in these approaches, the challenge remains to come out with the compounds that are broadly acceptable, safe, cost-effective, and reversible. In this regard, some plants, as well as chemical compounds, have been recognized for their value in the field of contraception. These compounds led to hypospermatogenesis *via* several effects, *viz.*, decrease in the testosterone level, disruption of the seminiferous tubules, and an increase in the oxidative stress. Though the intake of antioxidants may swamp the effects generated by these compounds [1–5], all other mechanisms contiguously affect the reproductive potential. The global scenario is now directing towards the use of alternative approaches which offer reversibility, efficacy, and safety with minimal side effects. So, the time has come to exploit the rich diversity of microorganisms since very little work has been done on their plausible application in this area. In the light of this initiative, previously in our laboratory, *Serratia marcescens* was found to produce a factor with sperm agglutinating property *in vitro* and showed admirable contraceptive efficacy in female mice [6]. These encouraging findings paved the way to extrapolate the same in male mice as a method of contraception. Also, due to the personal, productivity, and societal toll taken by surgery, a dire need continues to exist for nonsurgical methods of fertility control that are consistent, affordable, and satisfactory. Henceforward, the present study was designed for evaluating the impact of the sperm agglutinating factor (SAF) on the reproductive potential of male mice *via* an intratesticular route of administration.

## 2. Materials and Methods

### 2.1. Experimental Animals

Sexually mature male Balb/c mice (5-6 weeks old, 25 ± 2 g) were used in the present study, and they were housed in polypropylene cages. The animals were fed with standard pellet food and water ad libitum, and standard laboratory conditions (12 : 12, dark : light cycle) were retained. All the experimental work has been executed in accord with the procedures affirmed by Institutional Animal Ethics Committee, Panjab University vide letter no. PU/IAEC/S/16/140. All the experiments were completed in concurrence with the guidelines of the Committee for the Purpose of Control and Supervision of Experiments on Animals (CPCSEA).

### 2.2. Microorganism

A standard strain of *Serratia marcescens* (*S. marcescens*) (MTCC-7641), causing sperm agglutination *in vitro*, was used in the present study [6].

### 2.3. Isolation and Purification of the Sperm Agglutinating Factor (SAF) from *S. marcescens*

The sperm agglutinating factor was isolated and purified from a 72 h old cell culture of *S. marcescens* by the method earlier standardized in the laboratory [6].

### 2.4. Matrix-Assisted Laser Desorption/Ionization Time of Flight (MALDI-TOF) of SAF

Processing of protein band, tryptic digestion, and peptide extraction were performed as described by Gupta and Prabha [7]. Sample for MALDI-TOF analysis was prepared using the dried droplet method. 1 *μ*L peptide solution (peptide extracts after tryptic digestion) and 1 *μ*L of a suitable matrix, for example, alpha-cyano hydroxycinnamic acid (HCCA) in 1 : 2 v/v of acetonitrile (ACN) : 0.1% TFA, were mixed nicely. 1 *μ*L of this mixture was spotted on a MALDI target plate and allowed to air dry at room temperature. Peptide calibration standard (BRUKER) was also prepared in the same way. The MALDI target plate was loaded into Ultraflex MALDI-TOF for subsequent peptide spectra acquisition and analysis. Laser power of 337 nm wavelength was used for ionization of the samples spotted on the target plate. Peptide peaks were calibrated with peaks obtained from the peptide calibration standard. After peptide spectra were obtained, MS analysis was carried out using Flex analysis software (v 2.2, BRUKER). Subsequent MS data analysis was carried out using Biotools software (v 2.2, BRUKER) and MASCOT search engine (Matrix Science) against the NCBI database.

### 2.5. Intratesticular Inoculation Procedure

Under surgical conditions, male Balb/c mice were inoculated with different concentrations of SAF, *viz.*, 10, 50, 100, 200, or 400 *μ*g, in the right testis under anesthesia of ketamine and xylazine. The scrotal skin was gently washed with saline solution and disinfected with isopropyl alcohol. The inoculum (20 *µ*l) was instilled into the right testis serving as test while the left testis served as control in all the experimental groups except in case of hematological parameters and serum level of testosterone, where mice administered with PBS in the right testis served as control in comparison to mice receiving SAF in the right testis which served as test. To check the effect of SAF, the animals were euthanized on day 3, 7, 14, 21, 30, 45, 60, and 90. The parameters evaluated include body weight profile, TSI (%), seminal parameters, hematological parameters, serum level of testosterone, lipid peroxidation, and histological changes.

### 2.6. Weight Profile and Tissue Somatic Indices (TSI %)

The body weight of mice from each group on the 1^st^ day of the experiment was considered as initial body weight, and the body weight of the mice on the last day of the experiment was considered as final body weight. Mice were euthanized by cervical dislocation on the respective day of sacrifice. Mice were necropsied and various reproductive and nonreproductive organs were removed and weighed aseptically. The TSI (%) (organ weight/body weight × 100) of reproductive (vas deferens, testes, and cauda) and nonreproductive (kidneys, liver, spleen, and bladder) organs were estimated.

### 2.7. Evaluation of Seminal Parameters

#### 2.7.1. Total Sperm Count

Three mice from each group were sacrificed on the respective day of sacrifice. Immediately after necropsy, each vas deferens from both sides (i.e., right (inoculated) side and left (uninoculated) side) was pulled out, placed in two glass plates containing freshly prepared, prewarmed 200 *µ*l of PBS (50 mM, pH 7.2), and spermatozoa were enabled to swim out into the PBS by mild teasing. Furthermore, 10 *µ*l of the sample was placed on a clean glass slide and analyzed under a light microscope at 400x magnification. Around eight to ten fields were examined, and the mean number of spermatozoa in each field was multiplied by 10^6^.

#### 2.7.2. Sperm Motility

10 *µ*l of the semen sample was placed on a warm slide and analyzed (400x) under a light microscope. The motile and nonmotile sperms were counted in eight to ten fields, and percentage of motile sperms was determined.

### 2.8. Hematological Parameters

Mice were divided into two groups, *viz.*, test group (receiving 400 *µ*g SAF) and control group (receiving PBS), with 3 mice in each group. Mice from each group were sacrificed on day 90, and blood samples from mice in both the groups were analyzed for red blood cells (RBCs) and white blood cells (WBCs) counts, hemoglobin (hb) concentration, and hematocrit. Serum was analyzed to measure the activities of alanine aminotransferase (ALT) and aspartate aminotransferase (AST) according to the method of Reitman and Frankel [8].

### 2.9. Testosterone Assay

Mice from both the control group (receiving PBS) and the test group (receiving 400 *µ*g SAF) were sacrificed on day 90, and serum level of testosterone was measured by ELISA using a commercial kit according to the manufacturer's directions. The sensitivity of the assay was 5 pg/ml with intra- and interassay coefficient of variations being 5.1% and 7.5%, respectively.

### 2.10. Lipid Peroxidation

Mice receiving 400 *µ*g SAF in the right testis were sacrificed on day 3 and 7, and the level of lipid peroxidation was determined by the method of Ohkawa et al. [9]. Briefly, 3.3 ml TBA reagent (0.2 ml of 8.0% SDS, 1.5 ml of 20% acetic acid, and 1.5 ml of 0.8% aqueous thiobarbituric acid and 0.1% of butylated hydroxyltoluene) was mixed with 0.2 ml tissue supernatant obtained after the homogenization of testes, and the mixture was boiled at 95°C in a water bath for 60 min. The solution was cooled and centrifuged at 2000 rpm for 10 min. The supernatant was then used for recording absorbance against blank (distilled water) at 532 nm.

### 2.11. Histological Analyses

Mice from each group receiving a different concentration of SAF (10, 50, 100, 200, or 400 *µ*g) were sacrificed on day 3, 7, 14, 21, 30, 45, 60, and 90. The reproductive and nonreproductive organs after extraction were evacuated and fixed in 10% formaldehyde for 24 h. After 24 h, the tissues were inserted in paraffin. The paraffin tissue segments of 4 mm were stained with hematoxylin-eosin. Furthermore, slides were seen at 400x magnification for any noteworthy changes.

## 3. Results

### 3.1. Isolation and Purification of SAF from *S. marcescens*

Isolation and purification of SAF were performed by the method already standardized in our laboratory. Briefly, SAF was precipitated with 60–80% saturation of ammonium sulphate. After dialysis, it was subjected to Sephadex G-200 column, and from column chromatographic pattern, it was observed that the agglutinating activity was present in the fractions 4–8 with a peak value in fraction 5. Furthermore, the bioactive fractions were pooled, concentrated, and applied to a DEAE cellulose column. The column was run using a step gradient of NaCl, and SAF could be eluted with PBS containing 0.6 M NaCl. The molecular weight of purified SAF obtained after ion-exchange chromatography was estimated to be ∼54 kDa by SDS-PAGE.

### 3.2. MALDI-TOF of Purified SAF

**Figure 1:**
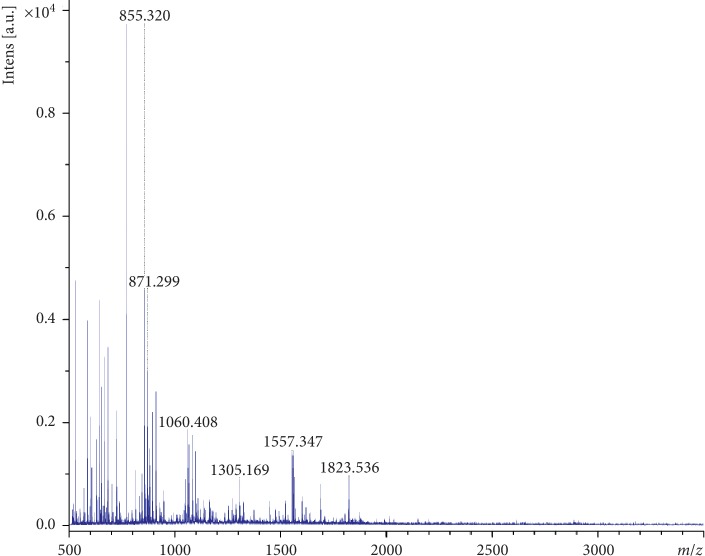
Mass spectrometric analysis of SAF after in-gel digestion with trypsin.

According to MALDI-TOF analysis of the purified 54 kDa band, the protein showed the peptide sequence similarity with adenylate kinase when the resulting spectrum was used to search for matching proteins in the NCBInr database, using the Mascot search program. The search yielded a top score of 56 for adenylate kinase (Figure 1).

### 3.3. Weight Profile

Single unilateral administration of different concentrations of SAF revealed no statistically significant changes in the body weight profile of male mice in all the groups (Supplementary [Supplementary-material supplementary-material-1]).

### 3.4. Tissue Somatic Indices (TSI %)

The TSI of reproductive and nonreproductive organs excised from all the mice receiving different concentrations of SAF were determined on all the days of sacrifice and the results so obtained indicated no significant changes in the TSI of all the organs (Supplementary Figures [Supplementary-material supplementary-material-1] and [Supplementary-material supplementary-material-1]).

### 3.5. Evaluation of Seminal Parameters

In order to assess treatment-related changes in seminal parameters, mice (*n* = 3) were euthanized on the respective day of sacrifice and sperm count and motility were assessed, and it was found that unilateral administration of SAF had sperm impairing effects on the right side of treatment only, whereas the left side showed no changes in seminal parameters. In the case of mice treated with 10 *μ*g of SAF, it was observed that the right side displayed azoospermia which persisted till day 7 only, whereas the left side displayed normal sperm count. Day 7 onwards the sperm count started to restore and on day 14 there was only a 20% decrease in the sperm count in the right side in comparison to the left side. Furthermore, on day 21 the sperm count of both the sides was comparable. Since complete inhibition of spermatogenesis on day 3 and 7 was observed, motility could not be assessed for the same, while on day 14 and 21 the motility in the right side was comparable to the left side (Table 1). In case of mice treated with a higher concentration of SAF, i.e., 50 *μ*g, azoospermia persisted in the right side up to day 14, whereas the left side showed normal sperm count. Thereafter, the sperm count started to restore, and on day 21 in the right side, there was only an 18% decrease in the sperm count in comparison to the left side. Furthermore, on day 30, the sperm count in the right side was comparable to the left side. In the case of motility, until day 14, motility could not be assessed due to complete inhibition of the spermatogenesis in the right side, while, on day 21, on the right side a 7% decrease was observed with respect to the left side, and it was comparable on day 30 (Table 1).

Mice treated with 100 *μ*g of SAF showed azoospermia in the right side till day 21 with respect to the left side which showed normal sperm count. On day 30, a 45% decrease in the sperm count was observed compared to the left side and sperm count started to restore thereafter, and on day 45 sperm count of the right side was comparable to the left side. Motility could not be assessed till day 21 due to complete inhibition of spermatogenesis in the right side. On day 30, a 30% decrease in the motility was observed on the right side in comparison to the left side (Table 1), and on day 45 motility of both sides was comparable. In case of mice treated with 200 *μ*g, azoospermia persisted in the right side up to day 45, whereas the left side showed normal sperm count. Thereafter, the sperm count started to restore, and on day 60 the sperm count on the right side was comparable to the left side. In the case of motility, until day 45, motility could not be assessed due to complete inhibition of the spermatogenesis in the right side, while, on day 60, motility was comparable on both sides (Table 1). With the concentration 400 *μ*g SAF, azoospermia could be achieved till day 90, whereas the left side displayed normal sperm count. Furthermore, on all the days, the motility could not be assessed due to complete inhibition of spermatogenesis in the right side (Table 1).

### 3.6. Hematological Parameters

No significant changes were found in the levels of AST (32.19 ± 3.12) and ALT (24.22 ± 0.45) in the treated mice compared to control mice AST (29.01 ± 3.01) and ALT (26.34 ± 0.19) in serum. Similarly, hematological parameters (haemoglobin, RBC, WBC, and hematocrit) in test (SAF 400 *µ*g) were comparable to the control (PBS) throughout the course of investigation (Table 2).

### 3.7. Testosterone Assay

A marked decrease was noted in the serum testosterone level after intratesticular administration of SAF (400 *µ*g) in the test (0.43 ± 0.02) as compared to the control (1.86 ± 0.03) receiving PBS (Table 2).

### 3.8. Lipid Peroxidation

**Figure 2:**
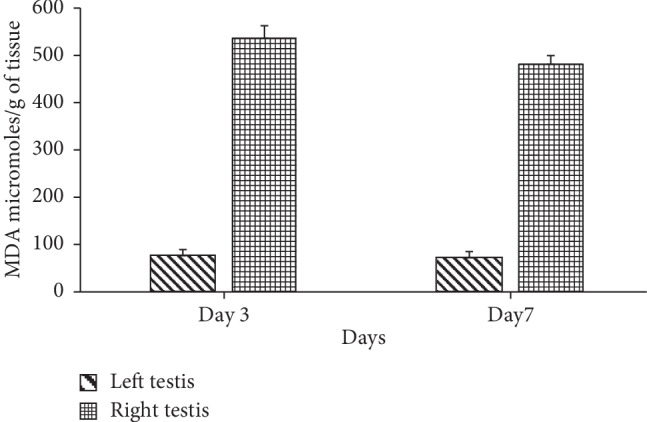
Lipid peroxidation in terms of MDA (*µ*moles/g of tissue) of testis in the right side in comparison to the left side on day 3 and 7. Data represent mean values ± SD. (^*∗*^), (^*∗∗*^), and (^*∗∗∗*^) represent *p* < 0.05, *p* < 0.01, and *p* < 0.001, respectively.

Following the single unilateral administration of 400 *µ*g SAF, a significant increase in endogenous thiobarbituric acid reactive substance (TBARS) levels was observed on day 3 and 7 in the right side (562.94 *µ*moles/g of tissue and 511.97 *µ*moles/g of tissue) as compared to the left side (65.38 *µ*moles/g of tissue and 74. 65 *µ*moles/g of tissue) (Figure 2).

### 3.9. Histological Examination

**Figure 3:**
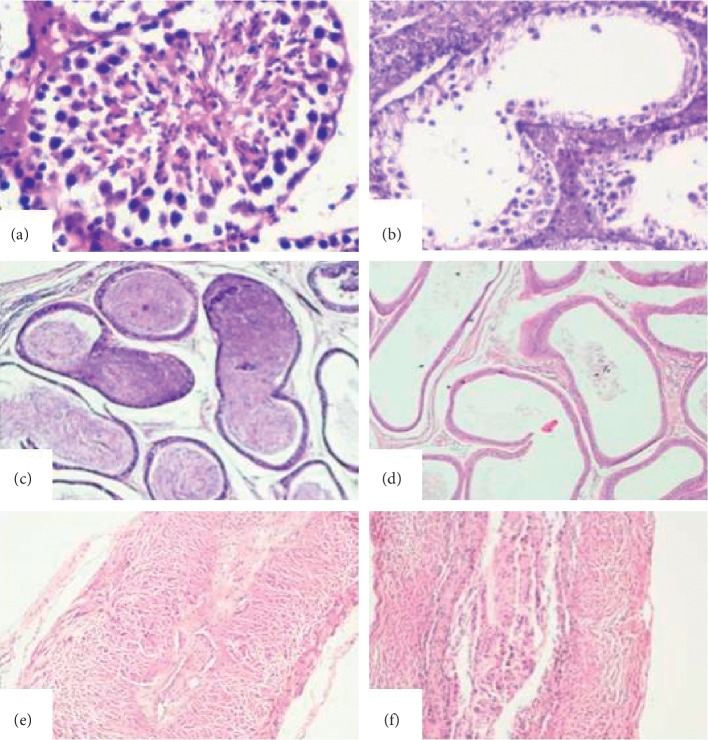
Representative photomicrographs of histological examination (day 90) of reproductive organs, *viz.*, testis, cauda epididymis, and vas deferens of left side (a, b, and c, respectively) and right side (d, e, and f, respectively) receiving 400 *µ*g SAF. *Left testis*: (a) normal seminiferous tubules (indicated by arrow head); *left cauda epididymis*: (b) tubules filled with spermatozoa (indicated by arrow head); *left vas deferens*: (c) normal tissue histology (abbreviated as N). *Right testis*: (d) degenerative changes in the seminiferous tubules and empty testicular lumen (abbreviated as ETL); *right cauda epididymis*: (e) empty epididymal lumen (abbreviated as EEL); *right vas deferens*: (f) normal tissue histology (abbreviated as N).

**Figure 4:**
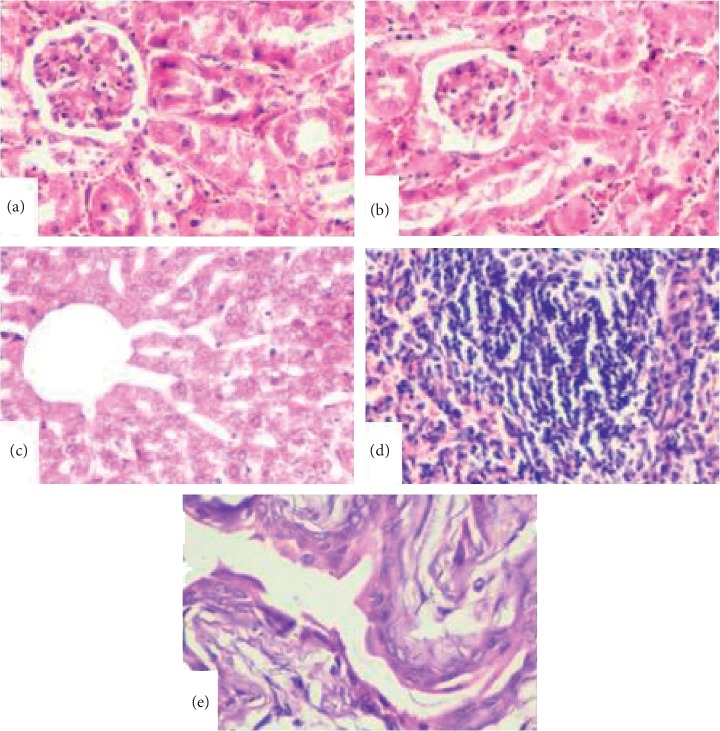
Representative histological photomicrographs (day 90) of various nonreproductive organs of male mice receiving 400 *µ*g SAF. (a) Left kidney. (b) Right kidney. (c) Liver (d) Spleen. (e) bladder.

To check any adverse impact of SAF on tissue morphology, histological analysis of different reproductive (testis, cauda epididymis, and vas deferens) and nonreproductive organs (spleen, kidney, bladder, and liver) was carried out. The right set of organs of mice treated with SAF (400 *μ*g) showed azoospermia with marked alterations in the histoarchitecture, *viz.*, loosening and sloughing of germinal epithelial in testes (Figure 3(d)), caudal epididymis showed empty tubules (Figure 3(e)), while vas deferens showed normal tissue histology (Figure 3(f)). However, the left set of reproductive organs divulged normal tissue histology, i.e., testis showed regular germinal epithelium and seminiferous tubules with progressive phases of change of spermatogonia into spermatozoa (Figure 3(a)), epididymis displayed filled tubules (Figure 3(b)), and vas deferens showed normal columnar epithelium (Figure 3(c)), while all the nonreproductive organs were observed to be histologically normal in all the cases (Figure 4).

## 4. Discussion

In the present study, we have made an attempt to demonstrate the effect of SAF on the reproductive potential of male mice by monitoring its effect on changes in body weight, TSI, hematological parameters, seminal parameters, testosterone level, lipid peroxidation, and tissue histology. The body weight profile of SAF-treated mice showed no significant changes and these findings are in line with the study by Reddy et al. [10] and Chauhan and Agarwal [11]; wherein, no change was reported in the body weight of the male rats treated with a microbial peptide nisin and extract of *Cassia fistula*. Furthermore, evaluation of TSI (%) of reproductive organs and nonreproductive organs was carried out to investigate their functional status ensuing various experimental conditions, and results of unilateral administration of SAF in right testis revealed no significant changes in %TSI. Furthermore, there was no significant difference in the hematological parameters, ALT, and AST in the treated group in comparison to the control. These results are in concordance with the studies where the antifertility effect of the extracts of *Coccinia indica*, *Mentha arvensis*, and a protein isolated from the root of *Achyranthes aspera* were evaluated. Thus, the absence of any changes in body weight, %TSI, and hematological parameters suggests the nontoxic nature of SAF and also indicates that it does not have an inimical effect on the general body metabolism.

Although various approaches for quantification of sperm quality have been suggested, seminal parameters have been most commonly used since high sperm count and motility are prerequisites for fertilisation and correlate strongly with fertilisation success. The results of the present study revealed complete loss of sperm count on the treated side after administration of 400 *µ*g of SAF. These results are in agreement with the studies carried out by Upadhyay et al. [12] who have shown that single unilateral administration of neem oil in male rats led to azoospermia in the side of treatment only.

There was a decrease in the testosterone level in the SAF-treated mice as compared to the control. It is well known that testosterone plays an important role in spermatogenesis and affects the epididymal milieu [13], so it must have affected the spermatogenesis. But, as hypospermatogenesis was seen only in the right side, there might be other factors playing an important role also. To understand the other possible mechanism by which SAF has affected the spermatogenesis, oxidative stress was checked as it is one of the most important factors affecting fertility by disturbing the spermatogenesis and steroidogenesis in testis. Due to the oxidative stress, free radicals are generated and these free radicals agitate the steroidogenic capacity of Leydig cells and differentiation of normal spermatozoa of germinal epithelium. Besides this, they also affect the cell membrane of spermatozoa because of the presence of a high amount of polysaturated fatty acids in their membrane, subsequently, prompting axonemal harm, decline in viability, and increase in morphological changes [14, 15]. So, the rise in the level of lipid peroxidation in the treated testis as compared to the nontreated side in the present study implies that the oxidative stress conditions in the treated side might have also played a crucial role in inhibiting the spermatogenesis and steroidogenesis.

To verify the effects of SAF, tissue histology was undertaken, where the right testis and right epididymis showed inhibition of spermatogenesis and empty epididymal tubules as compared to the left side where normal tissue histology was observed. The right testis showed significant degenerative changes in the seminiferous tubules and the changes brought about by the SAF in seminiferous tubule were uniform. The changes observed in the seminiferous tubules were intraepithelial vacuolization, loosening of germinal epithelium, and exfoliation of the germ cells. These results are in accordance to earlier studies where similar results were obtained when male mice were treated with gossypol tetra acetic acid and the leaf extracts of *Azadirachta indica*, *Allamanda cathartica*, *Curcuma longa*, *Dalbergia sissoo*, and SC12937 [16–21]. These degenerative changes in the seminiferous tubules of the right testis can be correlated to the azoospermia observed on the right side only while investigating the seminal parameters. As the unilateral administration of SAF affected only the reproductive organs of right side, it can be assumed that the inhibition of spermatogenesis was not mediated by any systemic mechanism as it would have otherwise affected the organs of both sides. In summary, intratesticular administration of SAF might be suppressing the spermatogenesis by affecting the steroidogenesis and by oxidative stress, which in turn leads to suppression of spermatogenesis, without eliciting any detectable toxic effects. Therefore, based on overall findings in the present study, it can be concluded that SAF holds the potential to be exploited as a male contraceptive in future.

## Figures and Tables

**Table 1:** Seminal parameters of male mice after intratesticular administration of SAF.

Concentration of SAF (*µ*g)	Days															
	Day 3				Day 7				Day 14				Day 21			
	Sperm count (×10^6^)		Motility (%)		Sperm count (×10^6^)		Motility (%)		Sperm count (×10^6^)		Motility (%)		Sperm count (×10^6^)		Motility (%)	
	L	R	L	R	L	R	L	R	L	R	L	R	L	R	L	R
10	15 ± 3.60	0	37.3 ± 2.30	0	17.3 ± 3.51	0	40.3 ± 4.02	0	14.6 ± 3.05	11.6 ± 2.08	53.3 ± 3.86	52.6 ± 3.08	15.6 ± 3.21	14.6 ± 1.52	38.3 ± 2.50	36.6 ± 1.15
50	15.6 ± 3.51	0	52 ± 2.55	0	14.6 ± 3.51	0	56.3 ± 4.72	0	12.6 ± 1.15	0	40.6 ± 3.05	0	20.3 ± 6.02	16.6 ± 1.52	43.6 ± 3.05	38.3 ± 2.77
100	22.3 ± 4.16	0	52 ± 4.1	0	17 ± 2	0	45 ± 5.66	0	23 ± 1	0	62 ± 3.05	0	15.3 ± 5.03	0	53 ± 5.02	0
200	21 ± 2.6	0	51 ± 5.03	0	18 ± 3	0	42 ± 5.08	0	19.6 ± 4.16	0	61 ± 6.05	0	18 ± 3	0	48 ± 5.07	0
400	20 ± 2.6	0	42.33 ± 4.13	0	32.3 ± 4.16	0	56.6 ± 2.05	0	26.6 ± 3.21	0	38.6 ± 3.05	0	28.3 ± 3.51	0	36 ± 5.32	0

Concentration of SAF (*µ*g)	Day 30				Day 45				Day 60				Day 90			
	Sperm count (×10^6^)		Motility (%)		Sperm count (×10^6^)		Motility (%)		Sperm count (×10^6^)		Motility (%)		Sperm count (×10^6^)		Motility (%)	
	L	R	L	R	L	R	L	R	L	R	L	R	L	R	L	R

10	ND	ND	ND	ND	ND	ND	ND	ND	ND	ND	ND	ND	ND	ND	ND	ND
50	19 ± 3.60	18.3 ± 4.16	43.6 ± 3.06	41.6 ± 2.05	ND	ND	ND	ND	ND	ND	ND	ND	ND	ND	ND	ND
100	17 ± 3.6	9.3 ± 2.51	46 ± 2.05	32 ± 3.5	18.6 ± 3.05	18.3 ± 2.08	41 ± 3.5	38 ± 5.08	ND	ND	ND	ND	ND	ND	ND	ND
200	15.6 ± 1.52	0	45 ± 5.3	0	14.6 ± 5.03	0	53 ± 5.4	0	20 ± 4.5	19.6 ± 3.51	57 ± 4.03	53 ± 3.50	ND	ND	ND	ND
400	32.3 ± 2.51	0	49.3 ± 6.05	0	21.6 ± 3.21	0	56.3 ± 3.05	0	18.3 ± 3.78	0	35.3 ± 4.15	0	24 ± 1.73	0	40.3 ± 3.05	0

(Data presented as mean ± SD; L: left side; R: right side; ND: not determined).

**Table 2:** Effect of intratesticular administration of SAF (400 *μ*g) on hematological parameters and hormonal levels of male mice.

Hematological parameters		
Parameters	Control (PBS)	SAF (400 *µ*g)
RBC (10^6^/mm^3^)	4.54 ± 0.21	4.72 ± 0.07
WBC (10^3^/mm^3^)	6.51 ± 0.31	6.02 ± 0.15
Hb (g/dl)	13.4 ± 0.02	13.9 ± 0.05
Lymphocytes (%)	78.16 ± 0.03	81.01 ± 0.01
Monocytes (%)	7 ± 0.18	5 ± 0.27
Neutrophils (%)	15 ± 0.09	13 ± 0.19
Eosinophils (%)	0.08 ± 0.04	0.03 ± 0.16
Basophils (%)	0.04 ± 0.21	0.07 ± 0.31
AST (*µ*/ml)	29.01 ± 3.01	32.19 ± 3.12
ALT (*µ*/ml)	26.34 ± 0.19	24.22 ± 0.45

Hormonal level		
Testosterone (ng/ml)	1.86 ± 0.03	0.43 ± 0.02

(Data presented as mean ± SD).

## Data Availability

The data used to support the findings of this study are included within the supplementary information files.

## Supplementary Materials

Supplementary Materials Supplementary Figure 1: body weight response of male Balb/c mice inoculated intravasally with SAF: (a) 10 *μ*g, (b) 50 *μ*g, (c) 100 *μ*g, (d) 200 *μ*g, and (e) 400 *μ*g. Supplementary Figure 2: tissue somatic indices (%) of various reproductive organs of mice administered with SAF: (a) 10 *μ*g, (b) 50 *μ*g, (c) 100 *μ*g, (d) 200 *μ*g, and (e) 400 *μ*g. Supplementary Figure 3: tissue somatic indices (%) of various nonreproductive organs of mice administered with SAF: (a) 10 *μ*g, (b) 50 *μ*g, (c) 100 *μ*g, (d) 200 *μ*g, and (e) 400 *μ*g.
