# Supplementary material for: Evaluation of Sperm Impairing Factor from Serratia marcescens as Male Contraceptive in Mouse Model
Source: Biomed Res Int. 2019 Oct 30;2019:9430964. doi: 10.1155/2019/9430964 (PMC6875308; doi:10.1155/2019/9430964)
Supplement: Supplementary Materials — Supplementary Figure 1: body weight response of male Balb/c mice inoculated intravasally with SAF: (a) 10 μg, (b) 50 μg, (c) 100 μg, (d) 200 μg, and (e) 400 μg. Supplementary Figure 2: tissue somatic indices (%) of various reproductive organs of mice administered with SAF: (a) 10 μg, (b) 50 μg, (c) 100 μg, (d) 200 μg, and (e) 400 μg. Supplementary Figure 3: tissue somatic indices (%) of various nonreproductive organs of mice administered with SAF: (a) 10 μg, (b) 50 μg, (c) 100 μg, (d) 200 μg, and (e) 400 μg. [file 9430964.f1.docx]

**SUPPLEMENTARY FIGURES**

**Suppl. Figure 1**

**
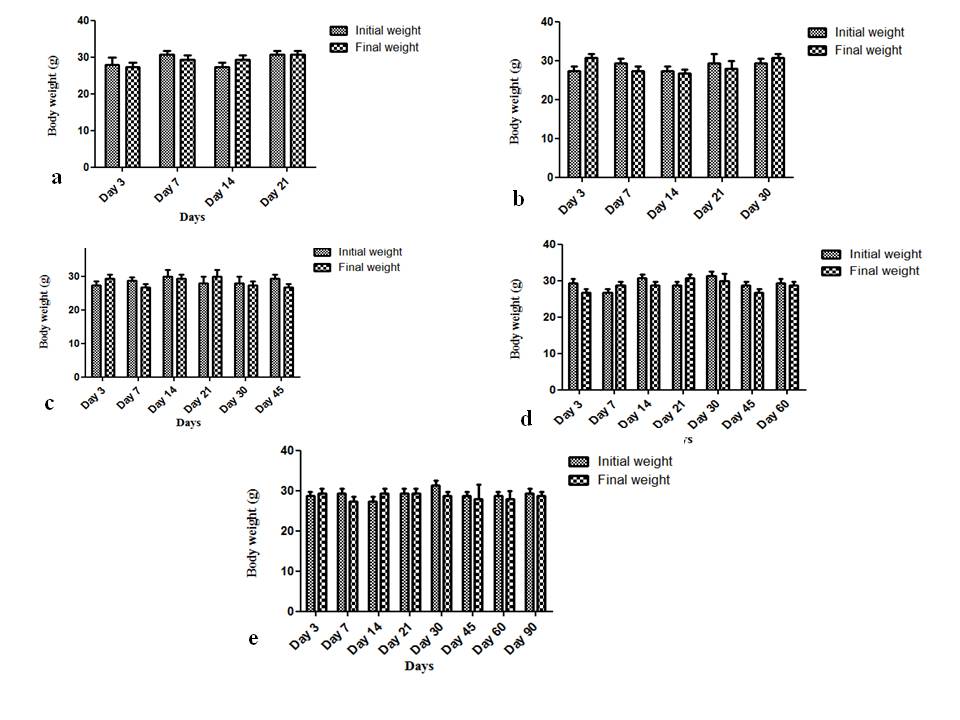
**

**Suppl. Figure 2**

**
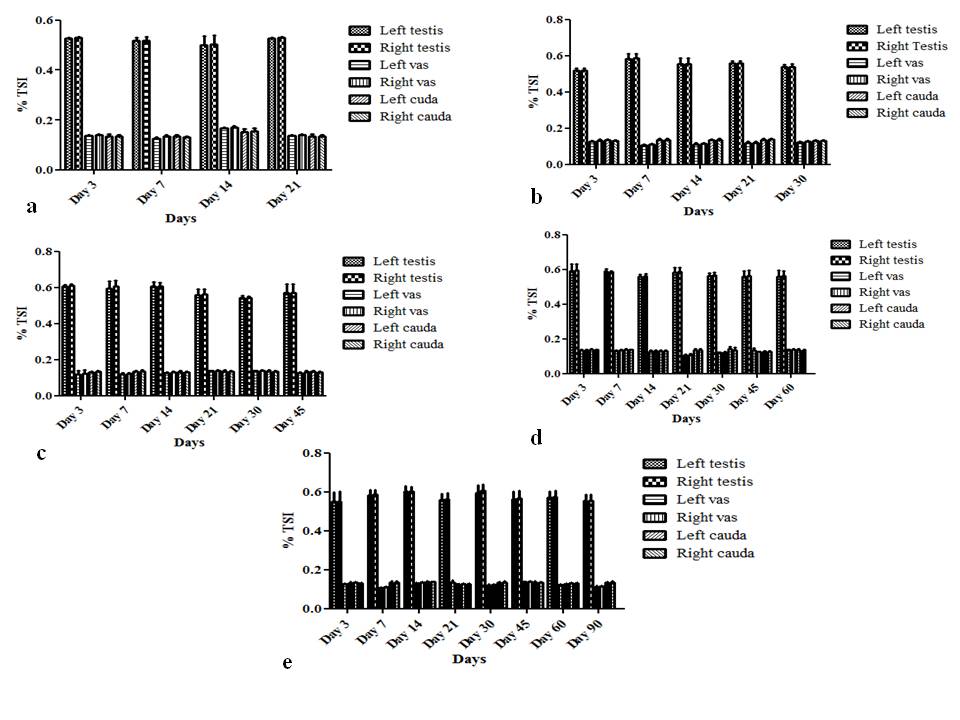
**

**Suppl. Figure 3:**

**
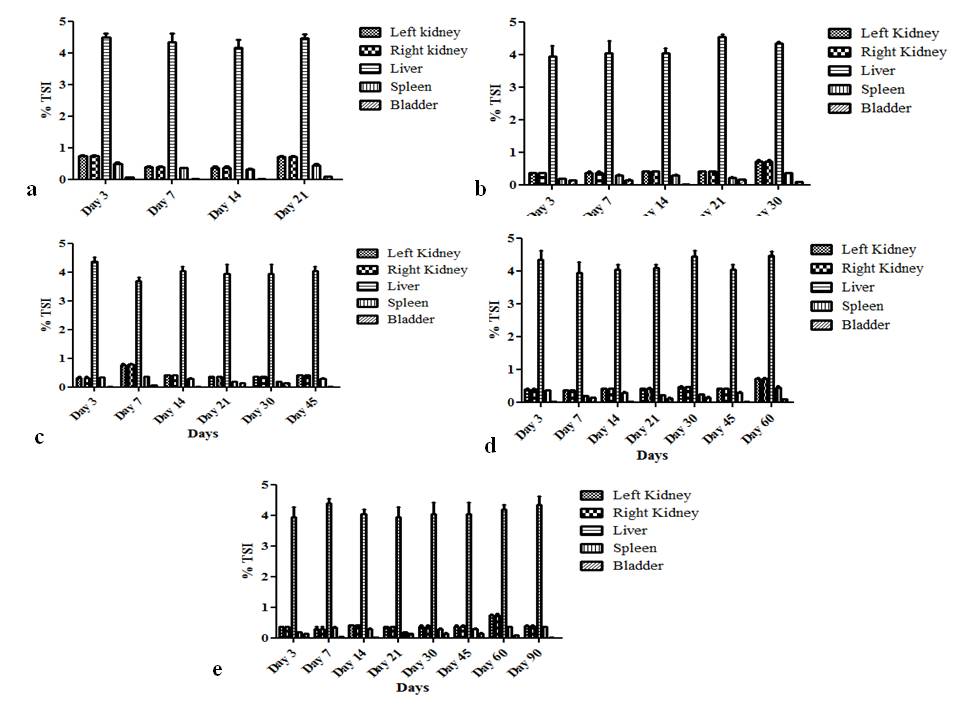
**

**Legends of Supplementary Figures**

**Suppl. Figure 1: Body weight response of male Balb/c mice inoculated intravasally with SAF a) 10μg b) 50μg c) 100μg d) 200μg e) 400μg**

**Suppl. Figure 2: Tissue somatic indices (%) of various reproductive organs of mice administered with SAF a) 10 μg b) 50 μg c) 100 μg d) 200** **μg e) 400 μg**

**Suppl. Figure 3: Tissue somatic indices (%) of various non reproductive organs of mice administered with SAF a) 10 μg b) 50 μg c) 100 μg d) 200 μg e) 400 μg**
